# To Collapse or not to Collapse Multiple Measures of the Same Psychological Construct: A Demonstration Across 14 Independent Studies of Autistic and Non-Autistic Children

**DOI:** 10.1007/s10802-026-01512-4

**Published:** 2026-10-14

**Authors:** Erin Reckner, Naveen Thourani, Drew Parker, John D. Herrington, Timothy P. L. Roberts, Robert T. Schultz, Brittany G. Travers, Chandan J. Vaidya, Ragini Verma, Birkan Tunç, Benjamin E. Yerys

**Affiliations:** 1 Center for Autism Research, Children’s Hospital of Philadelphia, 2716 South Street, 5th Floor, Philadelphia, PA 19146 USA; 2 Department of Psychology, University of Maryland-College Park, College Park, MD USA; 3 Department of Radiology, Diffusion and Connectomics in Precision Healthcare Research Lab, University of Pennsylvania, Philadelphia, PA USA; 4 Department of Child and Adolescent Psychiatry and Behavioral Science, Children’s Hospital of Philadelphia, Philadelphia, PA USA; 5 Department of Psychiatry, Perelman School of Medicine, University of Pennsylvania, Philadelphia, PA USA; 6 Advancing Transition and Learning for Adult Success Center, Department of Child and Adolescent Psychiatry and Behavioral Science, Children’s Hospital of Philadelphia, PA Philadelphia, USA; 7 Department of Psychology, Georgetown University, Washington, D.C. USA; 8 Occupational Therapy Program in the Department of Kinesiology, University of Wisconsin-Madison, Madison, Wisconsin USA; 9 Waisman Center, University of Wisconsin–Madison, Madison, Wisconsin USA; 10 Department of Radiology, Program in Advanced Imaging Research and Lurie Family Foundations MEG Imaging Center, Children’s Hospital of Philadelphia, Philadelphia, PA USA; 11 Department of Radiology, Perelman School of Medicine, University of Pennsylvania, Pennsylvania, PA USA

**Keywords:** Harmonization, Psychometrics, Convergent validity, Generalized linear model, Multi-site analysis, Big data

## Abstract

Large data repositories provide a valuable opportunity to combine multiple independent studies, creating large samples that can address questions not achievable within a single study. However, independent studies often use different instruments to measure the same behavior or psychological construct, such as cognitive ability, social functioning, or psychopathology. Investigators often combine samples that include different instruments under the assumption that instruments assessing the same construct capture the same underlying phenomenon. Yet this assumption of instrument interchangeability may be wrong. Collapsing different instruments into a single variable could introduce bias. Here, we established a quantitative method to demonstrate compatibility among multiple instruments with moderation analyses. We illustrate this method in domains of cognitive ability, anxiety symptoms, and attention-deficit/hyperactivity disorder symptoms as exemplars that might interact with outcomes of interest (autistic features instruments). A total of 1,352 participants were included from autistic and non-autistic samples across 14 independent studies. Our findings suggest that within this sample, some commonly used instruments within a domain can be acceptably combined, whereas others demonstrate discrepant relationships with outcomes of interest. More importantly, we offer a generalizable approach for scientists seeking to leverage existing datasets for secondary analyses.

**Supplementary Information:**

The online version contains supplementary material available at https://doi.org/10.1007/s10802-026-01512-4.

## Introduction

A primary challenge to mental health research is identifying the precise relationship between brain imaging data and clinical phenotypes. Most studies are limited by small samples (Button et al., 2013; Yarkoni, 2009). Complex brain and behavior relationships are likely small effects that require thousands of participants to identify reliable and meaningful results (Marek et al., 2022). One path to address the gap is completing coordinated, large-scale multi-site studies, such as the Adolescent Brain Cognitive Development Study (Casey et al., 2018) or a very large single-site study like the Philadelphia Neurodevelopmental Cohort (Calkins et al., 2015; Satterthwaite et al., 2014; Satterthwaite et al., 2016) that collect cognitive and mental health phenotype data and magnetic resonance imaging (MRI) scans at a scale that is not typically available for most studies. These approaches are effective for studying neurocognitive development but require a significant investment from a funder and study coordination by project leaders.

An alternative and cost-effective approach to generating a large-scale dataset is aggregating data from multiple small-scale, single-site studies. Research in the field of autism has produced two well-known efforts of such data aggregation: The National Database for Autism Research (Hall et al., 2012) and the Autism Brain Imaging Data Exchange (Di Martino et al., 2014). The biggest challenge in post-hoc data aggregation is harmonizing behavioral and cognitive data from multiple independent studies that were conducted without knowledge of the future aggregation. Data harmonization, as defined by Cheng et al., (2024), refers to “the practice of combining different datasets to maximize their comparability or compatibility” (pp. 1). While attention has been given to combining or harmonizing MRI data (Fortier et al., 2016; Fortin et al., 2017, Shen et al., 2025), behavioral and cognitive phenotypic data are often assumed to be comparable across studies with little to no quantitative assessment demonstrating their comparability. A common strategy is to combine different instruments that are believed to assess the same construct. For example, investigators may choose to administer different measures to estimate intellectual ability within their studies, such as the Differential Abilities Scales, 2nd Edition, Wechsler Intelligence Scales for Children, 4th Edition (now 5th Edition), or Wechsler Abbreviated Scales of Intelligence, 2nd Edition (Elliott, 2007; Wechsler, 2003, 2011). When merging data across studies with these various cognitive instruments, scientists may collapse the Full-Scale IQ scores, 2- and 4-subtest estimate Full-Scale IQ scores, and the General Conceptual Ability scores from these measures into a singular “Full-Scale IQ” variable. While this approach is common (Bertollo & Yerys, 2019; Cola et al., 2022; Granader et al., 2014; Pugliese et al., 2015; Ratto et al., 2018; Yerys et al., 2019), there is limited empirical data supporting it as an appropriate strategy other than psychometric testing showing correlations between cognitive assessments among children hovering around *r* = 0.8 (Sattler, 2018). One concern with this collapsing approach is that, even if the instruments are moderately correlated, they may relate differently to one’s outcome of interest. For example, if various intellectual ability instruments have demonstrably distinct relationships to measures of social function in autistic individuals, then employing this collapsing approach in control variables for brain–behavior analyses related to social function may distort the outcomes. If our goal with big data is to elucidate relationships between brain and behavior measures, then we would not want these relationships to be biased by our collapsing approach. This concern exists for collapsing both primary behavior outcome measures (e.g., social function measures), as well as commonly controlled variables that interact with outcome measures (i.e., cognitive ability).

Prior studies addressed this harmonization issue through advanced statistical modeling with item-level data or meta-analytic tools (van den Heuvel et al., 2020; Penberthy et al., 2005). The item-level analyses are elegant in their use of item-response theory and factor analysis to derive harmonized latent traits of a construct (e.g., memory). However, item-level data are not always available in aggregated datasets such as the Autism Brain Imaging Data Exchange (Di Martino et al., 2014). Meta-analytic tools are ideal when the same instrument or nearly identical instruments are used across studies. For example, combining multiple measures of attention deficit hyperactivity disorder (ADHD) can be easier with meta-analytic tools that take advantage of measures based on the 18 diagnostic symptoms of ADHD (e.g., ADHD Rating Scale (DuPaul et al., 1998), Vanderbilt ADHD Diagnostic Parent Rating Scale (Bard et al., 2013)). Unfortunately, many tools do not sample all 18 diagnostic symptoms, such as the Child Behavior Checklist (Achenbach et al., 2001). Thus, there is a critical need to develop analytic tools that harmonize phenotypic measures for which only aggregate data and scores are available, or the scales are not easily aligned at the level of individual items. By developing robust harmonization methods for diverse phenotypic measures of a construct, scientists can have confidence that collapsing multiple measures will not bias the results.

Consider a hypothetical study that will probe brain and behavioral relationships with aggregated data from *N* different sites to understand autistic features. All sites use the same measures, including the Social Responsiveness Scale (SRS-2), quantifying autistic traits; the Autism Diagnostic Observation Schedule, 2nd Edition (ADOS-2), assessing observed autistic behaviors; and Vineland Adaptive Behavior Scales (VABS)-Socialization Scale, capturing social autistic features. In contrast, the sites had a wide variety of instruments measuring cognitive ability, anxiety, or attentional problems. To control for the impact of these cognitive and co-occurring mental health factors on brain and behavior relationships related to autistic features, researchers may want to collapse the cognitive instruments into a single variable, as well as collapse relevant clinical instruments (e.g., anxiety or ADHD measures) into respective single variables. However, if various measures of cognitive functioning, anxiety, or ADHD have systematically different relationships with the autistic features outcome measures, then this collapsing approach could lead to biased results when included in brain-behavior analyses as control variables.

Then the question is, should the researchers in this hypothetical study collapse those various instruments or not? Here, we addressed this exact question. We evaluated potential bias in collapsing various instruments of IQ, anxiety symptoms, and ADHD symptoms as they relate to autistic features instruments in a large-scale study that aggregated data from many smaller-scale studies in a post-hoc manner. The aggregated data comes from the Connectomic Heterogeneity in Autism Research through Multi-site dMRI harmonization (CHARM) project (5R01MH117807-05) (Shen et al., 2025). Studies of autistic and non-autistic children were our test case for this method. However, this method is applicable for any analysis of large, post-hoc aggregated datasets.

## Methods

### Participants

1,352 participants between the ages of 4 and 42 were included in analyses (Table 1). sample predominantly consists of a pediatric age range (*N* = 1,282), with some studies recruiting individuals 18 + years of age (*N* = 70). 812 participants had a diagnosis of autism spectrum disorder, and 540 were classified as non-autistic (Non-ASD). Data were obtained across 14 studies from 7 independent study sites ([1] Children’s Hospital of Philadelphia (CHOP)); [2] Georgetown University; [3] University of Wisconsin-Madison. (UW-Madison); Autism Brain Imaging Data Exchange (ABIDE) sites: [4] New York University, [5] Barrow Neurological Institute (BNI), [6] San Diego State University, [7] Trinity College Dublin. BNI and CHOP contributed a study of individuals up to age 25, and another study from CHOP recruited autistic adults ages 25 to 42. 44 participants were included with Full Scale IQ < 70 (See Table S1 for breakdown by site).

**Table 1:** Sample description

	Non-ASD				ASD			
	*N*	Mean	SD	Range	*N*	Mean	SD	Range
Age in Years	540	12.34	4.38	3–42	812	11.65	3.73	4–36
Full Scale IQ	518	113.75	15.35	67–155	751	102.11	19.85	47–165
Non-verbal IQ	485	114.45	15.78	73–165	700	101.54	20.61	31–161
Verbal IQ	485	110.19	15.29	62–160	710	102.94	19.15	31–166
ADOS CSS	165	1.88	1.68	1–8	701	7.24	2.11	1–10
Vineland Social Standard Score	359	109.62	14.27	79–160	489	75.90	13.19	37–138
SRS-2 T-Score	494	43.98	5.02	37–63	710	72.90	9.87	46–109
CBCL Attention T-Score	188	52.29	3.42	50–70	278	65.75	9.70	50–97
BASC Attention T-Score	81	45.15	9.49	33–73	98	59.82	7.99	38–74
ADHD Rating Scale	211	5.24	5.10	0–30	267	23.85	11.75	0–51
SCARED Total Score	218	5.42	5.08	0–25	267	19.77	12.87	0–60
CBCL Anxiety T-Score	151	52.07	4.24	50–72	221	61.97	8.60	50–80
BASC Anxiety T-Score	81	43.79	7.08	31–64	98	55.78	13.30	31–85

Reported IQ includes DAS-II, WASI-II, WISC-IV, KBIT-2, & WISC-V

### Measures

*Autistic Features Measures.* [1] Social Responsiveness Scale: Second Edition (SRS-2, Constantino & Gruber, 2012) is a validated 65-item informant report, 4-point Likert scale rating of autistic traits. Higher scores indicate more social difficulties. SRS-2 Total T-scores were used for analysis (standardization sample mean (M) = 50; standard deviation (SD) = 10). [2] Autism Diagnostic Observation Schedule (ADOS): The ADOS (Lord et al., 2000) is a validated, semi-structured observational assessment designed to elicit autistic behaviors to inform diagnosis. We used the 10-point calibrated severity score rating (Range = 1–10) (Gotham et al., 2009; Hus & Lord, 2014). [3] The Socialization Domain from the Vineland Adaptive Behavior Scales-2nd, Edition (VABS; Sparrow et al., 2016) is an informant interview or report of adaptive social functioning on a 3-point scale (0 (never), 1 (sometimes), and 2 (usually or often)). This scale captures social functioning that occurs without prompting or assistance. For this analysis, the Socialization domain (VABS-Socialization) Standard Score was used (M = 100 and SD = 15).

*Cognitive Ability Measures*. [1] The General Conceptual Ability score was taken from the Differential Ability Scale, Second Edition (DAS-II; Elliott, 2007). [2] The 4-test or 2-test Full Scale IQ score, whichever was reported by the study, was taken from the Wechsler Abbreviated Scale of Intelligence, Second Edition (WASI-II; Wechsler, 2011). [3] The Full-Scale IQ score was taken from the Wechsler Intelligence Scale for Children, Fourth Edition (WISC-IV; Wechsler, 2003). Cognitive ability scores from other measures (KBIT-2, (Kaufman & Kaufman, 2004; WISC-5; Wechsler, 2014) are included in reported sample characteristics (Table 1); however, these instruments were not included in the cognitive ability harmonization due to insufficient sample size. All cognitive measures used standard scores where the standardization sample M = 100 and SD = 15.

*Anxiety and ADHD Measures.* [1] Screen for Child Anxiety Related Disorders (SCARED; Birmaher et al., 1997) is a 41-item scale that captures total childhood anxiety symptoms. Total raw scores were included in analyses (Range = 0 to 82). [2] Behavior Assessment System for Children - Second Edition (BASC; Reynolds & Kamphaus, 2006) is a multidimensional measure of clinical and adaptive behavior and personality. For this study, the anxiety (BASC-ANX) and attention (BASC-ATTN) subscales were included in the analyses. T-scores were used for analysis (standardization sample mean (M = 50; SD = 10)). [3] The Child Behavior Checklist (CBCL; Achenbach, 1999) is an instrument measuring behavioral/emotional problems. For this analysis, the attention problems (CBCL-ATTN) and anxiety problems (CBCL-ANX) subscales are included in analyses. T-scores were used for analysis (M = 50; SD = 10). [4] ADHD Rating Scale for Children and Adolescents (ADHD-RS; DuPaul et al., 2016) measures the frequency and severity of all 18 ADHD behaviors in youth with a 0 (Never/Rarely) to 3 (Very Often) score. Total raw scores were included in analyses (Range = 0 to 54).

### Analysis

We used linear ordinary least squares regressions to identify whether the relationship between score (IQ, attention, anxiety) and multiple reference measures of social behavior is significantly moderated by the instrument type. Each regression model included a ‘collapsed’ domain score (IQ, anxiety, or ADHD) as the dependent variable, a binary variable for coding the specific instrument type (e.g., 0 = DAS-II; 1 = WASI-II; see Table 2), a variable for the reference social measure (outcome of interest), and an interaction term for the instrument type and the reference social measure. For anxiety and ADHD domains, some summary variables were standardized scores, and others were raw scores. The SCARED and the ADHD Rating Scale provided raw score counts, and these scores were not adjusted in primary analyses. However, in supplemental analyses, we extracted residuals for age (Table S3). A significant interaction term in predicting the dependent variable indicated that the included instruments were non-equivalent in their relationship with the reference measure and should not be combined within a study using that reference measure as a variable of interest. For example, if a ‘collapsed’ IQ score was significantly predicted by the interaction of our reference social measure (e.g., Social Responsiveness Scale, 2nd Edition) and instrument type, we would not have combined scores from those IQ instruments into a single variable for analyses examining social skills with the Social Responsiveness Scale.

Regression models also included common control variables of age, gender, and diagnosis. The final model was:

$$\begin{array}{l} Domain\;Score\sim\;Covariates+Instrument\;Type\\+Autistic\;Features\;Reference\;Measure+Instrument\;Type\;*\;Autistic\\Features\;Reference\;Measure \end{array}$$

We ran models in a pairwise manner, each time comparing two given instrument types, and against a single reference measure at a time. For example, if we had three IQ instruments (three possible pairs) and three reference measures, we ran a total of nine models (3 pairs x 3 reference measures) for deciding whether IQ instruments should be collapsed or not. Samples for the two instrument types were matched for age, sex, and diagnosis before any regression model fitting. We used standardized betas as our indicators of effect size. The same methods were employed for IQ, anxiety, and ADHD instruments. Full model statistics are found in Tables 2, 3 and 4. See supplemental materials for analyses with FDR-corrected p-values (Table S2), analyses with age-regressed instruments (Table S3), analyses with the ASD-only sample (Table S4-6), as well as a comparison table for all analyses (Table S7).

**Table 2:** Linear model results for social reference measures and cognitive ability instrument interaction

	DAS-II & WASI-II	DAS-II & WISC-IV	WASI-II & WISC-IV
SRS-2	*n* = 191 *p* = 0.84 β = 0.04	*n* = 406 *p* = 0.71 β = -0.08	*n* = 290 *p* = 0.75 β = 0.07
ADOS	*n* = 304 *p* = 0.38 β = 0.13	*n* = 364 *p* = 0.58 β = -0.08	*n* = 282 *p* = 0.21 β = 0.19
VABS-Social	*n* = 272 *p* = 0.03 β = 0.62	*n* = 194 *p* = 0.72 β = 0.13	*n* = 166 *p* = 0.94 β = 0.03

*p* *≤* 0.05 indicates that interaction between instrument type and autistic features reference measure is significant; therefore, IQ instruments cannot be combined acceptably. β refers to the standardized beta of the interaction between instrument type and reference measure

## Results

### Cognitive Ability Instruments

Our analyses indicated that the DAS-II and WISC-IV were acceptable to combine when evaluating our autistic features instruments in our sample (Table 2). Instrument type did not significantly interact with any of the autistic features measures to predict the cognitive ability score: the SRS-2 (β=-0.08; *p* = 0.07), ADOS (β = -0.08; *p* = 0.58), or VABS-Social (β = 0.12; *p* = 0.72). Further, the WASI-II and WISC-IV were acceptable to combine: the SRS-2 (β = 0.07; *p* = 0.75), ADOS (β = 0.19; *p* = 0.21), and VABS-Socialization (β = 0.03; *p* = 0.94).

The DAS-II and WASI-II were acceptable to combine for the ADOS and SRS-2, as the instrument type and interaction with both the ADOS (β = 0.13; *p* = 0.38) and SRS-2 (β = 0.04; *p* = 0.84) did not predict the cognitive ability assessment score. For this sample, the DAS-II and WASI-II were not acceptable to combine for one autistic features measure, the VABS-Socialization, as the interaction term significantly predicted the cognitive ability assessment score (β = 0.62; *p* = 0.03); however, this interaction did not remain significant after FDR correction (Table S2). When these analyses were conducted in an ASD-only sample, similarly, DAS-II and WASI-II could not be combined; however, the pattern of significant interactions with autism features measures differed, with the ASD-only sample demonstrating a significant interaction with the SRS-2 (β = 1.55; *p* < 0.01). Further, in the ASD-only sample, the WASI-II and WISC-IV could not be combined due to a significant interaction with the SRS-2 (β = 1.35; *p* = 0.03) (Table S3).

### Anxiety Instruments

For anxiety, the SCARED and BASC-ANX, as well as the CBCL-ANX and BASC-ANX were acceptable to combine when evaluating all three autistic features measures in our sample (Table 3). Across the six regressions, standardized betas for the interaction terms ranged from − 0.17 to 0.28, and all p-values were greater than 0.11. Our analyses showed that we could not combine the SCARED and CBCL-ANX when evaluating autistic features with the SRS-2 or VABS-Socialization, because of significant interaction terms (β = -0.15; *p* < 0.01) and (β = 0.19; *p* = 0.02), respectively. These results remained significant after FDR correction (Table S2) and when age-related variance was extracted from the SCARED (Table S3). The results for the SCARED and BASC-ANX held in the ASD-only sample; however, in the ASD-only sample the CBCL-ANX and the BASC-ANX could not be combined due to a significant interaction term with the ADOS as the autistic features measure (β = 0.68; *p* = 0.03) (Table S5).

**Table 3:** Linear model results for social reference measures and anxiety instrument interactions

	SCARED & CBCL-ANX	SCARED & BASC-ANX	CBCL-ANX & BASC-ANX
SRS-2	*n* = 574 *p* < 0.01 β = -0.15	*n* = 352 *p* = 0.11 β = -0.15	*n* = 352 *p* = 0.08 β = 0.28
**ADOS**	*n* = 298 *p* = 0.66 β =-0.05	*n* = 192 *p* = 0.11 β = -0.17	*n* = 192 *p* = 0.82 β = -0.04
**VABS-Social**	*n* = 440 *p* = 0.02 β = 0.19	*n* = 348 *p* = 0.25 β = 0.13	*n* = 280 *p* = 0.57 β = -0.14

*p* ≤ 0.05 indicates that interaction between instrument type and autistic features reference measure is significant; therefore, anxiety instruments cannot be combined acceptably. β refers to the standardized beta of the interaction between instrument type and reference measure

### ADHD Instruments

For ADHD, the ADHD Rating Scale and CBCL-ATTN as well as the CBCL-ATTN and BASC-ATTN were not acceptable to combine when evaluating autistic features in our sample (Table 4). For the instrument pair of the ADHD Rating Scale and CBCL-ATTN, the interaction terms were significant with small to medium effects for regressions with SRS-2 (β = 0.26; *p* < 0.01), and VABS- Socialization (β = -0.25; *p* < 0.01). Results for the ADHD Rating Scale and CBCL-ATTN remained significant after FDR correction and after extracting age-residuals from the ADHD Rating Scale. These results did not replicate in the ASD-only sample, as the ADHD Rating Scale and CBCL-ATTN were acceptable to combine (Table S6). In the primary analysis, for the instrument pair of the CBCL-ATTN and BASC-ATTN, the interaction term was significant with small-to-medium effects for SRS-2 (β = 0.26.; *p* = 0.05) and ADOS (β = 0.31; *p* = 0.03). Results did not hold after FDR correction and were not replicated in the ASD-only sample (Table S6). In the primary analysis, for the instrument pair of the ADHD rating scale and the BASC-ATTN, the instrument type did not have a significant interaction term with any autistic features instruments. Across the three regressions, the interaction terms’ standardized betas ranged from − 0.16 to 0.09; and all p-values were greater than 0.27. These two instruments were deemed acceptable to collapse into a single ADHD variable for evaluating autistic features in our sample. However, for results with extracted age-residuals from the ADHD rating scale, the interaction term for VABS-Socialization was significant (β = 0.14.; *p* = 0.04).

**Table 4:** Linear model results for social reference measures and attention instrument interactions

	ADHD & CBCL-ATTN	ADHD & BASC-ATTN	CBCL-ATTN & BASC-ATTN
SRS-2	*n* = 654 *p* < 0.01 β = 0.26	*n* = 352 *p* = 0.27 β = 0.86	*n* = 352 *p* = 0.05 β = 0.26
ADOS	*n* = 318 *p* = 0.97 β < 0.01	*n* = 192 *p* = 0.86 β = 0.02	*n* = 192 *p* = 0.03 β = 0.31
VABS-Social	*n* = 496 *p* < 0.01 β = 0.25	*n* = 348 *p* = 0.07 β = 0.15	*n* = 300 *p* = 0.15 β = -0.27

*p* ≤ 0.05 indicates that interaction between instrument type and autistic features reference measure is significant; therefore, attention instruments cannot be combined acceptably. β refers to the standardized beta of the interaction between instrument type and reference measure

## Discussion

We sought to develop a quantitative approach for demonstrating that two instruments of the same construct are acceptable to collapse into a single variable. This method is needed at a time when public data repositories aggregating brain and behavior data include multiple independent studies of children from various clinical, neurodevelopmental, and typically developing populations. Researchers are motivated to maximize their sample size by collapsing data across these independent studies, even when studies use different measures to assess the same phenotypic features, such as cognitive ability, anxiety, or ADHD symptoms. When combining different instruments to bolster sample sizes, it is crucial that researchers ensure that individual instruments do not interact with variables of interest.

Here, we used a hypothetical scenario of wanting to examine brain-behavior correlations related to autistic features in an autistic sample with a desire to collapse across cognitive and co-occurring psychopathology instruments. Our simple yet effective moderation method with summary-level variables demonstrated that most cognitive and psychopathology instruments had non-significant interactions with our autistic features outcomes of interest. If we were evaluating brain-behavior relationships of autistic features, then we could have collapsed most of the cognitive and psychopathology instruments as control variables for our sample. However, we also identified a few significant interactions among anxiety instruments, which means we would not collapse the DAS-II and WASI-II, SCARED and CBCL Anxiety subscale, CBCL-Attention subscale and BASC Attention subscale, nor CBCL Attention subscale and ADHD Rating Scale for our hypothetical analysis of brain-behavior relationships for autistic features. Our findings of which instruments were acceptable and not acceptable to collapse were limited to our specific sample. However, this method can be applied to any aggregated dataset where only summary variables are available.

In addition, researchers should consider factors beyond statistical significance when determining the compatibility of collapsing two instruments. For example, researchers can reference the effect size of the interaction term as another indicator of whether it is “acceptable to combine” two instruments of the same construct. We contend that small effect sizes for interaction terms may indicate that the impact on the outcomes of interest may be negligible and not influence the larger effect sizes observed between brain-behavior correlations or the effect size between diagnostic groups. Contextual factors must be considered at the discretion of each researcher when determining collapsibility by referencing statistical significance and effect size.

For the CHARM sample, we identified that the DAS-II can be acceptably collapsed with scores from the WISC-IV into a single variable of cognitive ability, and the WAIS-II can be acceptably collapsed with scores from the WISC-V. However, the WASI-II was deemed unacceptable to combine with the DAS-II based on statistically significant interaction effects and the effect sizes. While the effect sizes for the interaction terms with the WASI-II were small to medium in our sample, the decision to combine distinct instruments of the same constructs was up to the hypothetical researcher’s discretion based on the context of the scientific question. Small inconsistencies between measures could introduce substantial bias; however, other research questions may not be impacted by small effect differences, and a researcher may decide that the WASI-II measure could be combined with the DAS-II or WISC-IV. It is essential to consider how complex modeling could interact with research variables that are negligibly but significantly different.

Another factor for researchers to consider is the number of reference measures used, and thus the number of reference measures that significantly interact with the instrument types. In analyzing cognitive ability in our hypothetical example, our effect sizes are small and potentially negligible when taken in isolation: the effect of instrument differences may be exacerbated in more complex models where the combined instrument variable interacts with other variables. For those cognitive instrument pairs deemed “not acceptable to combine,” this was a result of a significant interaction with one of the three autistic features instruments we used as a reference measure. Given the effect size was small for our hypothetical example of combining WASI-II and DAS-II with the Vineland’s Socialization scale as the reference measure, a scientist could determine to combine the WASI-II and DAS-II given the context of the research question. However, another investigator conducting multivariate analyses of small effects may determine that the small effects between the two cognitive measures and the Vineland could have wide-ranging impacts on their analysis. Additionally, some results replicated in the ASD-only sample, while other results did not, or some instrument pairs were deemed not acceptable to combine within the ASD-only sample even though they were acceptable in the full sample. This mixed replication is further indication that our approach does not generalize beyond the tested sample. In other words, if one’s initial sample is deemed acceptable to collapse a variable (e.g., anxiety measures) but you want to explore analyses in a sub-sample (e.g., ASD-only), then one must re-run the moderation analysis within the ASD-only sample.

For anxiety instruments in our sample, we found that the SCARED and CBCL Anxiety scores could not be acceptably collapsed into a single variable for all autistic features measures, whereas scores from the SCARED and BASC Anxiety subscale as well as the CBCL Anxiety and BASC Anxiety subscale can be acceptably combined for all autistic features measures. For ADHD instruments in this sample, the ADHD Rating Scale and the CBCL attention subscale, as well as the CBCL and BASC respective attention subscales, could not be collapsed into a single variable, whereas the ADHD Rating Scale and BASC attention scale could be acceptably combined. In our analyses, raw scores and age-adjusted residuals yielded the same results. However, we encourage investigators to examine age-adjusted or sex-adjusted residuals in moderation analyses, as age, sex, or other characteristics may influence the phenotypic raw scores. The significant interactions within the anxiety and attention instruments were small to moderate effect sizes across multiple reference measures. Given the interactions’ effect sizes, a researcher may consider not combining these measures, as instrument type could impact the results when the key outcome is one of our autistic features measures.

Overall, our results highlight that researchers should be vigilant in examining such instrument type differences when considering collapsing across instruments of the same construct. Researchers should consider the effect size of significant instrument interactions within the context of the scientific aims and research questions. Prior to using this moderation approach, researchers should carefully review the original source study’s inclusion criteria and test battery to confirm the appropriateness of measures used to ensure that the source data was validly collected. We believe our study has broad implications, because we established a quantitative method for scientists to demonstrate compatibility among any set of phenotypic instruments purported to capture the same psychological construct. Our approach allows researchers to make statistically informed decisions about combining data across instruments.

Our methods can be applied to datasets beyond CHARM, including datasets within the National Institute of Mental Health Data Archive, the Autism Brain Imaging Data Exchange, and the Reproducible Brain Charts (Shafiei et al., 2025), because all these repositories consolidate brain and behavior data across numerous independent research studies. Large-scale repositories like these could benefit from our methods to improve the reliability of behavior measurements tapping into the same latent construct when probing brain-behavior relationships, genomic associations, or even complex relations among multiple behavior domains. Scientists could also use our approach when collapsing variables that will serve as the outcome/dependent variable and use other independent or control variables as the reference measures. Regardless of how researchers would use our methods, researchers who implement our data consolidation methods can merge datasets together with confidence that doing so would not bias their analyses. Thus, our phenotype integration methods are a foundational tool to foster robust moderation methods across neuroscience and psychiatric research.

Future research should establish whether our findings are generalizable to other clinical populations or cognitive instruments. Our present cohort was limited by only including cross-sectional data, and thus we were unable to explore whether these methods can demonstrate the ability to collapse versions of the same instrument that may be used in longitudinal analyses. Further, site effects were confounded with the type of instrument administered (e.g., one site used the DAS-II, whereas a second site used the WASI-II), and the nature of each site’s recruited sample (e.g., including or excluding intellectually disabled participants or specific age ranges), therefore, no independent term for site was included in our model. Another limitation was the lack of validation for the use of some measures with autistic youth. Future work should explore full psychometric properties of these commonly used instruments in autistic youth. In addition, future research can test whether the results from this analysis hold across different autism samples. Addressing these limitations will improve the applicability of these methods across the broader psychiatric and neuroscience contexts.

In this use case of collapsing instruments across samples of autistic populations, we used common instruments of autistic features as our reference measures of interest. However, our approach can be replicated across any population with multiple measures of the same construct, offering a rigorous method to minimize instrument bias and enable robust, large-scale secondary data analyses across the field of child and adolescent psychopathology.

## Supplementary Information

Below is the link to the electronic supplementary material.

Supplementary Material 1

## Data Availability

The data that support the findings of this study are derived from multiple sources. Data from the Autism Brain Imaging Data Exchange (ABIDE) are publicly available. Additional datasets are subject to data use agreements and are not publicly available but may be accessed upon reasonable request and with permission from the respective institutions.
