# Supplementary material for: To Collapse or not to Collapse Multiple Measures of the Same Psychological Construct: A Demonstration Across 14 Independent Studies of Autistic and Non-Autistic Children
Source: Res Child Adolesc Psychopathol. 2026 Oct 14;54(5):118. doi: 10.1007/s10802-026-01512-4 (PMC13668954; doi:10.1007/s10802-026-01512-4)
Supplement: Supplementary file 1 [file 10802_2026_1512_MOESM1_ESM.docx]

Supplemental Materials for “To collapse or not to collapse multiple measures of the same psychological construct: a demonstration across 14 independent studies of autistic and non-autistic children.”

| **Table S1**  Number of IQ < 70 by Site | |
| --- | --- |
| **Site** | **IQ < 70, n** |
| **ABIDEII-BNI** | 0 |
| **ABIDEII-NYU** | 0 |
| **ABIDEII-SDSU** | 1 |
| **ABIDEII-TCD** | 0 |
| **CHOP** | 40 |
| **Georgetown** | 0 |
| **UW–Madison** | 3 |

| **Table S2**  FDR Corrected P-Values | | | | | | |
| --- | --- | --- | --- | --- | --- | --- |
|  | **SRS-2** | | **ADOS** | | **VABS-Social** | |
|  | *p* | *p.adj* | *p* | *p.adj* | *p* | *p.adj* |
| DAS-II & WASI-II | 0.84 | 0.84 | 0.38 | 0.57 | 0.03 | 0.09 |
| DAS-II & WISC-IV | 0.71 | 0.72 | 0.58 | 0.72 | 0.72 | 0.72 |
| WASI-II & WISC-IV | 0.75 | 0.94 | 0.21 | 0.63 | 0.94 | 0.94 |
| SCARED & CBCL-ANX | 0.00 | 0.03 | 0.66 | 0.66 | 0.02 | 0.03 |
| SCARED & BASC-ANX | 0.11 | 0.165 | 0.11 | 0.165 | 0.25 | 0.25 |
| CBCL-ANX & BASC-ANX | 0.08 | 0.24 | 0.82 | 0.82 | 0.57 | 0.82 |
| ADHD & CBCL-ATTN | 0.01 | 0.02 | 0.97 | 0.97 | 0.01 | 0.02 |
| ADHD & BASC-ATTN | 0.27 | 0.41 | 0.86 | 0.86 | 0.07 | 0.21 |
| CBCL-ATTN & BASC-ATTN | 0.05 | 0.08 | 0.03 | 0.08 | 0.15 | 0.15 |
| *Note*. *p* indicates original analyses p-value while *p.adj* refers to the FDR adjusted p-values. | | | | | | |

| **Table S3**  Linear model results for autism features reference measures and anxiety and attention instrument interactions with age residual extracted from SCARED and ADHD Rating Scale. | | | | |  |
| --- | --- | --- | --- | --- | --- |
|  | **SCARED & CBCL-ANX** | **SCARED & BASC-ANX** | **ADHD &**  **CBCL-ATTN** | **ADHD &**  **BASC-ATTN** | |
| **SRS-2** | n = 574  *p* < 0.01  β = 0.12 | n = 352  *p* = 0.11  β = -0.12 | n = 652  *p* < 0.01  β = 0.20 | n = 352  *p* = 0.28  β = 0.06 | |
| **ADOS** | n = 298  *p* = 0.67  β =-0.04 | n = 192  *p* = 0.11  β = 0.14 | n = 318  *p* = 1.00  β < 0.01 | n = 192  *p* = 0.92  β = 0.01 | |
| **VABS-Social** | n = 440  *p* = 0.01  β = 0.16 | n = 348  *p* = 0.21  β =0.11 | n = 496  *p* < 0.01  β = 0.22 | n = 348  *p* = 0.04  β = 0.14 | |
| *Note.* p ≤ 0.05 indicates that interaction between instrument type and reference measure is significant; therefore, anxiety instruments cannot be combined acceptably . β refers to the standard beta of the interaction between instrument type and reference measure. | | | | |  |

| **Table S4**  Linear model results for social reference measures and cognitive instrument interactions in ASD sample. | | | |
| --- | --- | --- | --- |
|  | **DAS-II & WASI-II** | **DAS-II & WISC-IV** | **WASI-II & WISC-IV** |
| **SRS-2** | n = 208  *p* < 0.01  β = 1.55 | n = 260  *p* = 0.75  β = 0.16 | n = 170  *p*=0.03  β = 1.35 |
| **ADOS** | n = 232  *p* = 0.86  β = 0.04 | n = 294  *p* = 0.11  β = 0.36 | n = 186  *p* = 0.28  β = 0.28 |
| **VABS-Social** | n = 148  *p* = 0.68  β = 0.22 | n = 130  *p* = 0.45  β = 0.51 | n = 98  *p* = 0.91  β =0.07 |
| *Note*. p ≤ 0.05 indicates that interaction between instrument type and reference measure is significant; therefore, cognitive ability instruments cannot be combined acceptably. β refers to the standard beta of the interaction between instrument type and reference measure. | | | |

| **Table S5**  Linear model results for social reference measures and anxiety instrument interactions in ASD sample | | | |
| --- | --- | --- | --- |
|  | **SCARED & CBCL-ANX** | **SCARED & BASC-ANX** | **CBCL-ANX & BASC-ANX** |
| **SRS-2** | n = 304  *p* = 0.24  β = -0.17 | n = 190  *p* = 0.59  β = -0.16 | n = 190  *p*=0.72  β = 0.17 |
| **ADOS** | n = 292  *p* = 0.95  β = 0.01 | n = 160  *p* = 0.37  β = -0.16 | n =160  *p* = 0.03  β = -0.68 |
| **VABS-Social** | n = 256  *p* = 0.03  β = 0.40 | n = 186  *p* = 0.71  β = -0.10 | n = 160  *p* = 0.91  β = -0.06 |
| *Note.* p ≤ 0.05 indicates that interaction between instrument type and reference measure is significant; therefore, anxiety instruments cannot be combined acceptably. β refers to the standard beta of the interaction between instrument type and reference measure. | | | |

| **Table S6**  Linear model results for social reference measures and attention instrument interactions in ASD Sample | | | |
| --- | --- | --- | --- |
|  | **ADHD & CBCL-ATTN** | **ADHD & BASC-ATTN** | **CBCL-ATTN & BASC-ATTN** |
| **SRS-2** | n = 334  *p* = 0.98  β < 0.01 | n = 190  *p* = 0.18  β = 0.36 | n = 190  *p*=0.15  β = 0.62 |
| **ADOS** | n = 292  *p* = 0.91  β = 0.01 | n = 160  *p* = 0.75  β = 0.05 | n = 160  *p* = 0.68  β = 0.12 |
| **VABS-Social** | n = 246  *p* = 0.75  β = 0.06 | n = 186  *p* = 0.75  β = 0.41 | n = 168  *p* = 0.41  β = 0.36 |
| *Note*. p ≤ 0.05 indicates that interaction between instrument type and reference measure is significant; therefore, attention instruments cannot be combined acceptably. β refers to the standard beta of the interaction between instrument type and reference measure. | | | |

| **Table S7**  Overview of results across primary and supplementary analyses. | | | | | | | | | | |
| --- | --- | --- | --- | --- | --- | --- | --- | --- | --- | --- |
|  |  | *Cognitive Ability* | | | *Anxiety* | | | *ADHD* | | |
|  |  | **DAS-II**  **&**  **WASI-II** | **DAS-II**  **&**  **WISC-IV** | **WISC-IV**  **&**  **WASI-II** | **SCARED**  **&**  **CBCL-ANX** | **SCARED**  **&**  **BASC-ANX** | **CBCL-ANX**  **&**  **BASC-ANX** | **ADHD-RS**  **&**  **CBCL-ATTN** | **ADHD-RS**  **&**  **BASC-ATTN** | **CBCL-ATTN &  BASC-ATTN** |
| SRS | Primary Analysis | Acceptable to Combine | Acceptable to Combine | Acceptable to Combine | Not Acceptable to Combine | Acceptable to Combine | Acceptable to Combine | Not Acceptable to Combine | Acceptable to Combine | Not Acceptable to Combine |
|  | FDR-Corrected | - | - | - | Result remains significant | - | - | Result remains significant | - | Result no long significant |
|  | ASD-Only Sample | Not Acceptable to Combine | Acceptable to Combine | Not Acceptable to Combine | Acceptable to Combine | Acceptable to Combine | Acceptable to Combine | Safe to  Combine | Acceptable to Combine | Acceptable to Combine |
|  | Age-Regressed Analysis | - | - | - | Not Acceptable to Combine | Acceptable to Combine | - | Not Acceptable to Combine | Acceptable to Combine | - |
| ADOS | Primary Analysis | Acceptable to Combine | Acceptable to Combine | Acceptable to Combine | Safe to  Combine | Acceptable to Combine | Acceptable to Combine | Safe to  Combine | Acceptable to Combine | Not Acceptable to Combine |
|  | FDR-Corrected | - | - | - | - | - | - | - | - | Result no long significant |
|  | ASD-Only Sample | Acceptable to Combine | Acceptable to Combine | Acceptable to Combine | Safe to  Combine | Acceptable to Combine | Not Acceptable to Combine | Safe to  Combine | Acceptable to Combine | Acceptable to Combine |
|  | Age-Regressed Analysis | - | - | - | Safe to  Combine | Acceptable to Combine | - | Safe to  Combine | Acceptable to Combine | - |
| VABS-Social | Primary Analysis | Not Acceptable to Combine | Acceptable to Combine | Acceptable to Combine | Not Acceptable to Combine | Acceptable to Combine | Acceptable to Combine | Not Acceptable to Combine | Acceptable to Combine | Acceptable to Combine |
|  | FDR-Corrected | Result no long significant | - | - | Result remains significant | - | - | Result remains significant | - | - |
|  | ASD-Only Sample | Acceptable to Combine | Acceptable to Combine | Acceptable to Combine | Not Acceptable to Combine | Acceptable to Combine | Acceptable to Combine | Safe to  Combine | Acceptable to Combine | Acceptable to Combine |
|  | Age-Regressed Analysis | - | - | - | Not Acceptable to Combine | Acceptable to Combine | - | Not Acceptable to Combine | Not Acceptable to Combine | - |
| *Note*. ‘Acceptable to Combine’ indicates that the interaction between the instrument pair and the autistic features reference measure is non-significant (*p* > 0.05). ‘Not acceptable to combine’ indicates that the interaction between the instrument pair and the autistic features reference measure is significant (*p* ≤ 0.05). ‘Result no longer significant’ indicates that the p-value from the primary analysis was no longer significant (*p* > 0.05) following FDR correction. ‘Result remains significant’ indicates that the p-values from the primary analysis remained significant (*p*≤0.05) following FDR Correction. The hyphen symbol (-) indicates that that analysis is not relevant in the context of those measures. Namely, only analyses involving the SCARED and ADHD-RS are reported for the age-regressed analysis, and FDR-corrected results are not reported for ‘Acceptable to Combine’ results from the primary analysis. See tables 2-4 for the full model statistics for the primary analysis. See Table S2 for FDR-corrected p-values. See Table S3 for the full model statistics for the age-regressed analysis. See Tables S4-S6 for full model statistics for the ASD-only sample. | | | | | | | | | | |

| **Table S8**  Instrument Availability by Site | | | | | | | | | | | | |
| --- | --- | --- | --- | --- | --- | --- | --- | --- | --- | --- | --- | --- |
| **Site** | **WISC-IV** | **WASI-II** | **DAS-II** | **SCARED** | **CBCL-ANX** | **BASC-ANX** | **ADHD-RS** | **CBCL-ATTN** | **BASC-ATTN** | **SRS** | **ADOS** | **VABS-Social** |
| **ABIDEII-BNI** | - | - | - | - | - | - | - | - | - | Yes | - | - |
| **ABIDEII-NYU** | - | - | Yes | - | - | - | - | - | - | Yes | Yes | Yes |
| **ABIDEII-SDSU** | - | Yes | Yes | - | - | - | - | - | - | Yes | Yes | - |
| **ABIDEII-TCD** | - |  | - | - | - | - | - | - | - | Yes | Yes | - |
| **CHOP** | Yes | Yes | Yes | Yes | Yes | Yes | Yes | Yes | Yes | Yes | Yes | Yes |
| **Georgetown** | - |  | - | - | Yes | - |  | Yes | - | Yes | Yes | - |
| **UW–Madison** | - | Yes | - | - | - | - | - | - | - | Yes | Yes | Yes |
